# Modulating gut microbial biofilms for intestinal barrier repair: opportunities in nutritional intervention

**DOI:** 10.3389/fnut.2026.1855344

**Published:** 2026-07-22

**Authors:** Yunhan Huang, Jiayao An, Tianyi Cui, Zhi Cui, Tingting Xia, Xin Zhao

**Affiliations:** 1 Ministry of Education Key Laboratory of Pharmacology of Traditional Chinese Medical Formulae, Tianjin University of Traditional Chinese Medicine, Tianjin, China; 2 State Key Laboratory of Chinese Medicine Modernization, Tianjin University of Traditional Chinese Medicine, Tianjin, China; 3 School of Medical Technology, Tianjin University of Traditional Chinese Medicine, Tianjin, China

**Keywords:** biofilms, gut microbiota, IBD, intestinal barrier, nutritional intervention

## Abstract

**Background:**

The gut microbiota has emerged as a key therapeutic target in clinical nutrition for special medical purposes for repairing intestinal barrier damage. However, current strategies primarily focus on modulating microbial composition while overlooking the functional microbial community structure—particularly biofilms, which play important roles in gut physical, chemical and immune barrier homeostasis.

**Methods:**

Relevant literature on nutrition intervention, gut microbiota and biofilm, and intestinal barrier published in PubMed and CNKI up to June 2026 was narratively reviewed.

**Results:**

This review systematically analyzes nutrients-biofilm-barrier axis, highlights the function of microbial biofilm formation and interaction involved in barrier integrity, and evaluates innovative nutritional interventions capable of modulating biofilm formation, including with enteral nutrition (EN) and parenteral nutrition (PN). Clinically, we discuss how this biofilm-centric approach offers enhanced clinical outcomes in managing barrier dysfunction associated with diseases such as IBD, compared to conventional microbiota modulation alone.

**Conclusion:**

The function of nutrients to effectively modulate the gut microbial biofilm in barrier integrity has been increasingly recognized in recent years. Future research is needed to further explore biofilm-based nutritional interventions and their therapeutic role in disease management.

## 1 Introduction

Targeting the gut microbiota has become an important approach within the realm of nutritional intervention to repair intestinal barrier (1, 2). Many chronic intestinal diseases (such as IBD, leaky gut) are closely related to the continuous damage to barrier function of pathogenic bacteria (3). Consequently, interventions based solely on strain profiles are likely to be incomplete. While strategies such as probiotics or prebiotics supplementation have shown efficacy in certain contexts, clinical outcomes exhibit substantial inter-individual variability, limiting their translational potential. In terms of clinical nutritional interventions, patients often receiving standardized formulations that do not account for individual variability. Thus, identifying more focused targets for microbiota intervention is essential to advancing the efficacy of clinical nutrition during intestinal barrier repair.

Gut microbial biofilms are well-documented structures prevalent in both health and disease and can adopt a biofilm lifestyle *in vivo* (4–6). Symbiotic biofilms are structured communities formed by microorganisms by secreting extracellular polymers (such as polysaccharides and proteins), and can enhance the viability and pathogenicity of bacteria (7). Abnormal colonization or structural damage of harmful bacteria can directly or indirectly damage the function of the intestinal barrier by biofilm formation (8). On the contrary, the formation of biofilm of beneficial bacteria has a certain protective effect on the intestinal barrier (9). Existing studies (10, 11) have primarily focused on validating whether specific nutrients regulate biofilm formation in particular bacterial species. Thus, there is an urgent need to systematically synthesize the role of biofilm as the central axis linking nutrients, gut microbiota, and host barrier.

Nutritional formulations can effectively modulate gut microbial biofilms to optimize nutrient matrices and enhance bioavailability in clinic, especially in Inflammatory Bowel Disease, IBD (12, 13) However, the microbial mechanisms underlying treatment responses remain unclear. To advance this paradigm, we introduce a conceptual framework—the “nutrients-biofilm-barrier axis”—which posits that certain nutrients (e.g., dietary fibers, polyphenols, amino acids) regulate bacterial quorum sensing systems, thereby altering biofilm architecture (thickness, porosity) and metabolite profiles (SCFAs, antimicrobial peptides, toxins), which in turn differentially impact intestinal barrier components (tight junction proteins, mucus layer, immune cells). We would like to further enables a stepwise clinical algorithm from nutritional risk screening to biofilm detection, barrier assessment, and targeted intervention, offering an actionable reference for more effective interventions.

## 2 How gut microbial biofilm impacts intestinal barrier integrity

The biofilm of the intestinal flora contains both the embedded multiple bacteria into the same biofilm matrix, and contains the biofilm formed independently of different flora and is in close contact in space (14). In multi-strain biofilms, different bacteria share the microenvironment through extracellular polysaccharides (EPS), exchange metabolites and quorum sensing signaling molecules (9). Neighboring independent biofilms achieve cross-biofilm interactions through physical contact or diffusible molecules (e.g., metabolites, antimicrobial peptides) (15). Given their structural and bacterial communication complexity, biofilms are not merely microbial aggregates but are critically interacted with the host’s intestinal mechanical barrier, chemical barrier, and immune responses.

### 2.1 Bacterial biofilm formation: producers, regulators, and crosstalk

The formation mechanism of intestinal bacterial biofilms varies depending on bacterial species characteristics and environmental signals (Tables 1, 2). Gut commensals such as *Bacteroides fragilis*, and *Lactobacillus* sp. could form biofilms in the intestine to promote bacterial adhesion, protect the intestine barrier against pathogens, and interaction with the host (16, 17, 151–153), while biofilms formed by *Akkermansia muciniphila*, *Bifidobacterium* sp., *Lactococcus* sp. and *Bacillus subtilis* could be detected *in vitro* (18–21). Gut pathogens such as species of *Pseudomonas*, *Klebsiella*, and *Staphylococcus* can also form biofilms to activate inflammatory and immune responses (22). The presence of biofilm of other general bacteria such as *Bacillus* and *Lactococcus* can elicit a host immune response (23, 24). Different bacterial groups affect the structure and function of biofilm through unique regulatory pathways (such as Gram-negative bacteria dependent quorum sensing system (QS) and c-di-GMP system, while Gram-positive bacteria are regulated by the LuxS system and Agr system), thus having completely different effects on the host intestinal barrier.

**Table 1:** Probiotic biofilms: formation and barrier-protective mechanisms.

Bacteria	Key biofilm genes	Biofilm structure	Barrier protection mechanisms	Nutritional modulation potential	References
*B. fragilis*	QS system: *luxR*	Dense, heterogeneous, polysaccharide-rich	↓Toxins	Inducing regulatory T cells (Tregs) to exert immunomodulatory functions	(16, 116–119, 151, 152)
*Lactobacillus*	c-di-GMP system	Dense but permeable	↑Antimicrobial peptides; ↑immune tolerance;↓inflammation	Dietary fiber, plant extracts	(17, 120–125)
*Bacillus subtilis*	epsA-O operon, tapA operon	Var*iable (surface/interface)*	↑Stress tolerance; host immune response	Not specified	(19, 126, 127)
*Lactococcus*	Not found	Not found	↑Transfer of biofilm-related genes; conjugation	Not specified	(128)
*Bifidobacterium*	QS system: dppC, livM, luxS, sapF	EPS-rich, porous	↑Stress response and polysaccharide metabolism; immune cell recruitment and cytokine production	FOS, GOS, HMOs	(18, 43, 129–135)
*A. muciniphila*	QS system	Loose, mucus-associated	↑Mucus turnover; host metabolism	Polyphenols, dietary fiber	(15, 20, 136, 137)

**Table 2:** Pathogenic biofilms: formation and barrier-disruptive mechanisms.

Bacteria	Key biofilm genes	Biofilm structure	Barrier disruption mechanisms	Nutritional modulation potential	References
*Pseudomonas*	QS system (las/rhl), c-di-GMP, cAMP-CRP; adhesins (LapA, HecA)	Alginate-rich, dense	↑Extracellular polysaccharides; ↑chronic inflammation, tissue damage, immune escape	Mannose/fructose; honey/propolis extract	(33, 138–142)
*Klebsiella*	luxR, luxS, c-di-GMP, type III pili	Thick capsule, drug-resistant	↑Capsular polysaccharides; ↑excessive inflammation, tissue necrosis, bacteremia	Plant polysaccharides	(143–148)
*Staphylococcus*	Agr QS system	Dense, adherent	↑Toxins (e.g., PSM); ↑inflammatory cytokines (IL-1β, TNF-*α*); ↓immune clearance	Lauric acid; nisin; green tea extract; caprylic acid	(149, 150)

**Figure 1:**
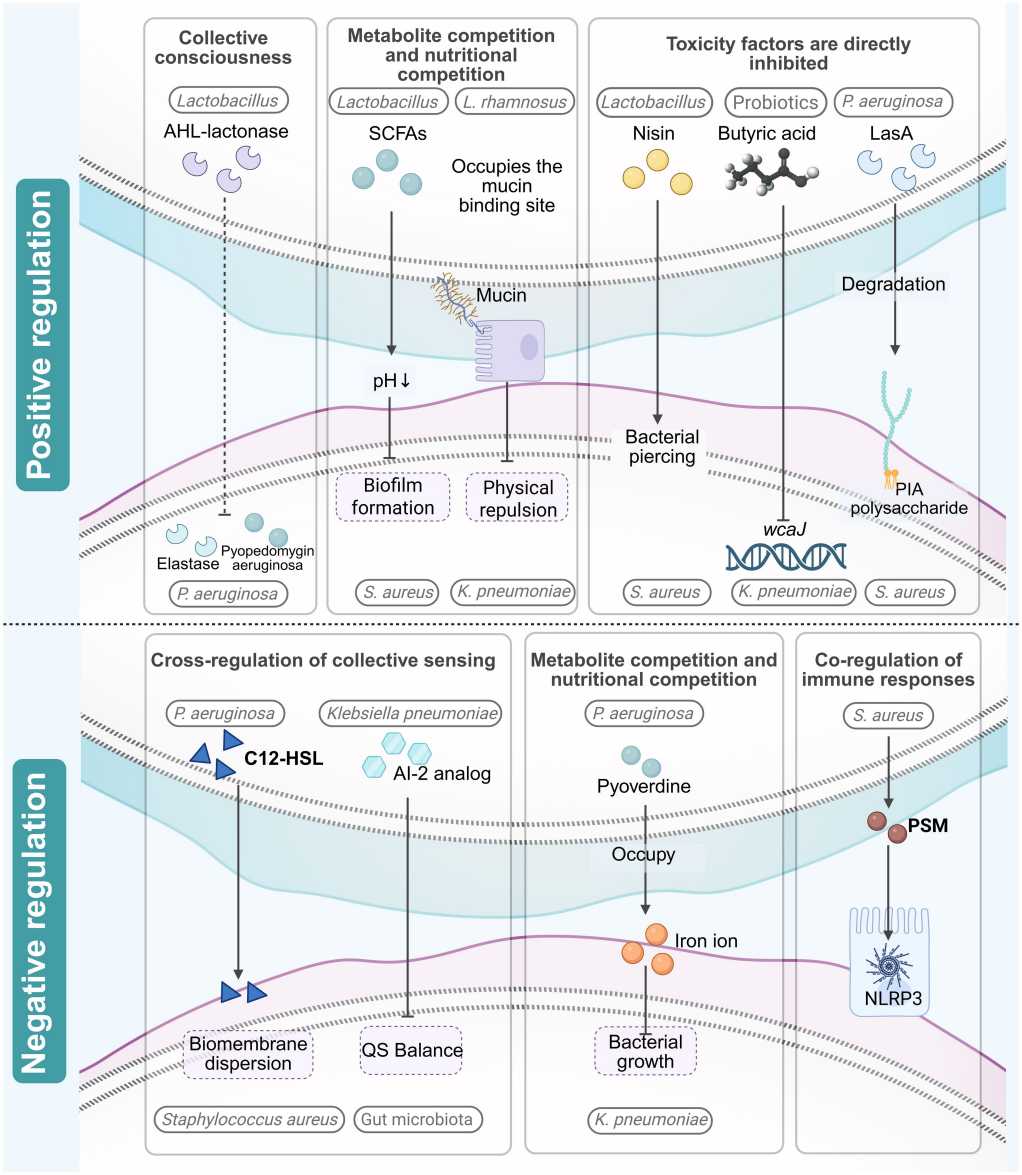
Regulatory crosstalk of microbial biofilm in shaping intestinal colonization. Schematic diagram illustrating positive and negative interspecies crosstalk among gut commensal and pathogenic biofilms, covering quorum sensing interference, metabolite competition, and immune co-regulation mechanisms. Created in https://BioRender.com.

The biofilm between different bacteria in the gut transmits information and thus affects the host. In positive regulation, probiotics work synergistically through multiple mechanisms: quorum sensing signal interference is manifested by *Lactobacillus* secreting AHL-lactonase, degrading Gram-negative bacterial signaling molecules and inhibiting virulence factors of *Pseudomonas aeruginosa* (25, 26); Metabolites compete with nutrients including *Lactobacillus* to produce SCFAs that lower environmental pH and inhibit biofilm formation of *Staphylococcus aureus* and *Klebsiella pneumoniae* (26–28). *L. rhamnosus* achieves physical exclusion by occupying mucin binding sites (29). Direct inhibition of toxic factors is manifested by the perforation of the cell membrane of *S. aureus* by streptococcitin and nisin (30). Butyric acid produced by commensal bacteria, inhibits the synthesis of the pathogen’s capsular polysaccharides, and the LasA enzyme produced by *P. aeruginosa* degrades the PIA polysaccharides of *S. aureus* (31, 32). Gut commensal biofilm activation of regulatory T cells could suppress excessive inflammation (33). Additionally, recent studies have confirmed that *A. muciniphila* exhibits anti-biofilm properties against pathogens and can form symbiotic biofilms in consortium with other commensals such as *Bacteroides ovatus* and *Bifidobacterium breve* (15, 20). In terms of negative regulation, pathogenic bacteria cross-regulate through quorum sensing, such as *P. aeruginosa* releasing C12-HSL to induce the spread of *S. aureus* biofilm (34), *K. pneumoniae* producing AI-2 analogs that interfere with the intestinal flora QS balance (35), and metabolic competition. For example, *P. aeruginosa* secretes pyocyanin to snatch iron ions to inhibit the growth of *K. pneumoniae* and aggravate oxidative damage to the host (36, 37). Additionally, *S. aureus* produces phenolic regulatory proteins that activate the NLRP3 inflammasome, promoting the invasion of *K. pneumoniae* and the exacerbation of pneumonia, to enhance its drug resistance and survival advantages (38, 39). This ecological perspective reveals that biofilms are not isolated entities but nodes in a complex network of interspecies interactions. Nutritional interventions can exploit these interactions—for instance, by providing substrates that favor beneficial cross-feeding networks while starving pathogenic consortia. Understanding the rules of this ecological game is essential for designing effective biofilm-targeted therapies. Complex prebiotic blends such as mixtures of inulin (40), as well as human milk oligosaccharides (HMOs) and resistant dextrins (41, 42) can be chosen to support diverse beneficial biofilm communities, promoting cross-feeding and metabolic cooperation that enhances overall ecosystem stability when beneficial biofilms are deficient. HMOs disrupt pathogen biofilm architecture and quorum sensing while selectively enriching *Bifidobacterium* (43). Polyphenols (e.g., cranberry extract, green tea catechins) can be selected to selectively inhibit pathogenic quorum sensing systems while sparing beneficial communication when pathogenic biofilms predominate (44, 45). Lactoferrin and lysozyme may be incorporated into enteral formulas when modulation of inter-species competition is needed, as they differentially affect pathogen and commensal biofilm formation (46). Symbiotic combinations—matching specific probiotic strains with carefully selected prebiotics—can be designed to engineer desired ecological outcomes based on the patient’s baseline biofilm profile. Thus, the choice of nutritional intervention should be tailored to the target biofilm ecosystem, applying subtractive strategies when pathogens dominate, and additive strategies like supporting beneficial networks when protective biofilms are deficient. This targeted selection of nutrients based on biofilm ecological assessment represents an individualized approach to modulating complex interspecies interactions within the gut microbiota (Figure 1).

### 2.2 Biofilm-facilitated SCFAs accumulation, antibacterial substances and toxins

Bacterial cross-feeding in the same biofilm matrix can promote the synthesis of short-chain fatty acids (SCFAs). For example, *Mitsuokella jalaludinii* can degrade phytic acid into the intermediate product 3-hydroxypropionic acid through a complete metabolic pathway, which can be converted into the propionic acid in synergy with *Anaerostipes rhamnosivorans* (47). SCFAs can promote the formation of *Bacillus subtilis* biofilm and then play the role of probiotics (48). Importantly, the biofilm structure reduces the diffusive loss of SCFAs (49). When dominant membranous bacteria (such as *Bifidobacteria*) decreases, the biofilm structure is loose and the SCFAs yield decreases, reduction in the thickness of the mucus layer. Dietary fiber interventions that enhance SCFA-producing biofilms are associated with improved barrier function in conditions like IBD (50). However, the clinical response depends on the baseline abundance of fiber-degrading bacteria capable of forming such biofilms, highlighting the need for personalized approaches. Dietary fibers (e.g., pectin, fructooligosaccharides, resistant starch) serve as fermentable substrates that promote the growth and biofilm formation of SCFA-producing bacteria such as *Faecalibacterium prausnitzii* and *Bifidobacterium* (51–53). Resistant dextrins increase SCFA production and lower luminal pH to inhibit pathogen biofilms, coupled with butyrate-driven immune regulation (54). These fibers enhance EPS production, stabilize biofilm architecture, and increase local SCFA concentrations at the mucosal surface. Acetate and butyrate, when added as postbiotics to enteral formulas, can further reinforce biofilm integrity and barrier function.

Bacteria produce antibacterial substances and enrich them, inhibiting the formation and colonization of other bacterial biofilms. For example, *Lactococcus lactis* secretes nisin, which can inhibit surrounding bacterial biofilms and enhance the competitive advantage of microecology (51). *Bacillus* can produce signal molecules, such as surfactants, which lead to changes in membrane potentials, triggering population sensing such as biofilm formation and spore formation in response to environmental stress (55). On the other hand, the expression of the biofilm formation-related gene *enoA1* of *Lactobacillus plantarum* activates host cells through pattern recognition receptors (TLRs/NOD2), promotes the secretion of antimicrobial peptide HBD-2, and enhances mucosal immunity (56). LuxS/AI-2-mediated population sensing system plays an important role in the synthesis of bacteriophageal NMD-17 in co-cultivated *L. plantarum* (57). Prebiotic fibers (e.g., inulin, GOS) stimulate beneficial bacteria to produce antimicrobial peptides (e.g., nisin from *Lactococcus lactis*) that are concentrated within biofilms (58). Certain amino acids (e.g., glutamine) enhance host antimicrobial peptide secretion via epithelial cell signaling. Plant-derived compounds (e.g., berberine, resveratrol) can upregulate bacterial bacteriocin production, strengthening the competitive advantage of beneficial bacteria biofilms against pathogens (59, 60). However, the efficacy of such interventions may be limited by the host’s ability to support biofilm formation, which is influenced by factors such as mucus composition and immune status.

Metabolites released by pathogenic bacterial biofilms can harm host health through multiple mechanisms. Pathogenic bacterial biofilms (such as sulfate reducing bacteria (SRB), *Escherichia coli*) metabolize to produce hydrogen sulfide (H_2_S) (61), lipopolysaccharide (LPS) (62) and other toxic substances. The biofilm matrix impedes its diffusion, leading to increased local concentrations. H_2_S induces oxidative stress in epithelial cells by inhibiting mitochondrial complex IV activity (63); The LPS immune response guides the Toll-like receptor 4 (TLR4) signaling pathway, promotes the release of pro-inflammatory factors such as TNF-*α*, IL-6, destroys the structure of tight junction proteins, causes damage to mucosal integrity, and inhibits the phagocytic function of macrophages (33). The local enrichment of toxins within pathogenic biofilms provides an explanation for the “hotspot” nature of mucosal lesions in IBD and the difficulty in eradicating infections with systemic antibiotics. Nutritional interventions that disrupt biofilm matrix integrity (e.g., certain enzymes or plant-derived compounds) could enhance the efficacy of conventional therapies by improving toxin diffusion and immune access (64). Sulfur-restricted diets reduce hydrogen sulfide production by sulfate-reducing bacteria within pathogenic biofilms. Plant-derived polyphenols (e.g., epigallocatechin gallate from green tea) disrupt biofilm matrix integrity, facilitating diffusion and clearance of accumulated toxins (65). Lactoferrin supplementation chelates iron, limiting pathogen metabolism and toxin production (66). Dietary antioxidants (vitamin C, vitamin E) neutralize oxidative damage caused by biofilm-released toxins (67).

### 2.3 Biofilm-mediated expression of tight junction proteins

Under the stimulation of external substances, the intestinal barrier function is in dynamic change, and tight junction proteins play an important role in maintaining intestinal barrier function (68). The biofilm formed by symbiotic bacteria (such as lactic acid bacteria) can directly bind to the surface receptors of intestinal epithelial cells, induce the aggregation and phosphorylation of tight junction proteins such as ZO-1 and occludin, and enhance intercellular adhesion (68, 69). Studies have found that encapsulation of EPS in biofilms provides several important advantages to cells (69–71). Furthermore, biofilm endosymbionts continuously release low-level stimulation signals through the TLR/MyD88 pathway, promote the expression of intestinal epithelial repair genes (such as REG3γ), while inhibiting the excessive activation of proinflammatory factors (72). These findings suggest that nutritional strategies aimed at strengthening beneficial bacteria biofilms (e.g., by providing specific prebiotic fibers that enhance EPS production) could offer a sustained approach to fortify the intestinal barrier in conditions characterized by increased permeability, such as leaky gut syndrome and IBD. Specific prebiotic fibers (e.g., resistant starch, beta-glucan) promote EPS production in beneficial bacteria biofilms, enhancing their physical stability and epithelial adhesion. Glutamine and arginine, when supplemented in enteral nutrition, directly support tight junction protein expression (ZO-1, occludin) and synergize with biofilm-derived signals (73, 74). Butyrate and other SCFAs, either produced endogenously or added as postbiotics, activate GPR109A receptors on epithelial cells to enhance junctional integrity (75).

Biofilms provide protective shells for pathogens to escape host defenses. For example, an increase in the number of adhesion-invasive *E. coli* forming biofilms is associated with the occurrence of ulcerative colitis (76). Studies have also found that biofilms formed by *Campylobacter jejuni* secrete specific proteases that directly cleave occludin, and other tight junction proteins (such as ZO-1) (76). *E. coli* biofilm could destroy the phosphorylation of ZO-1 by secreting toxins, increasing intestinal mucosa permeability (77). Nutritional strategies that inhibit the expression of virulence factors (e.g., by interfering with quorum sensing) could serve as adjunctive therapies to protect epithelial integrity (78). Mannose-rich oligosaccharides competitively inhibit type I fimbriae-mediated adhesion of pathogenic *E. coli*, preventing biofilm formation on epithelial surfaces (79). Cranberry proanthocyanidins block P-fimbriae binding, reducing pathogen colonization (44). Butyrate supplementation downregulates expression of proteases that cleave tight junction proteins (80). Zinc carnosine strengthens epithelial junctions and counteracts protease-mediated damage (81).

### 2.4 Biofilms shape anti-inflammatory immune responses

Gut commensal biofilms can induce macrophages to produce more anti-inflammatory cytokines, such as IL-10 and TGF-*β*, thereby suppressing inflammatory responses (82). *Bacteroides thetaiotaomicron* and *F. prausnitzii*, two metabolically complementary bacteria, regulate the intestinal mucus barrier through modification of goblet cells and mucin glycosylation. This process reinforces mucosal immune defense and facilitates the establishment of intestinal epithelial and immune homeostasis in the colon (83). The immunomodulatory properties of beneficial biofilms underpin the therapeutic potential of probiotics and prebiotics in inflammatory disorders. However, the inter-individual variability in baseline Treg/Th17 balance may explain why some patients respond better to such interventions, pointing toward the need for immune profiling prior to nutritional therapy (84, 85). Dietary tryptophan is metabolized by biofilm-associated bacteria (e.g., *Lactobacillus*) into indole derivatives that activate AhR signaling, promoting Treg differentiation (86, 87). Polyphenols (e.g., quercetin, resveratrol) modulate macrophage polarization toward M2 phenotype when delivered via biofilm-enriched microenvironments (88, 89). Diet and microbiota-derived SCFAs, particularly butyrate, enhance Foxp3 expression and regulatory T cell expansion (90). Zinc and selenium, as micronutrient cofactors, support anti-inflammatory immune programming (91).

The LPS released by the biofilm of Gram-negative bacteria enters the circulatory system through membrane vesicles, activates the TLR4 pathway of monocytes, induces the large release of cytokines such as IL-6 and TNF-*α*, triggers increased permeability of intestinal mucosal vascular and neutrophil infiltration, resulting in an increase in the apoptosis rate of epithelial cells (33, 62). Pathogenic biofilms (e.g., Enterobacteriaceae) influence the NOD2 pathway by secreting flagellin, leading to sustained damage to the intestinal mucosa in chronic inflammation (e.g., IBD) (92). The persistent immune activation driven by pathogenic biofilms is a key factor in the chronicity of IBD. Nutritional interventions that suppress biofilm formation (e.g., through dietary exclusion of specific pro-biofilm nutrients) could help break the cycle of inflammation and tissue damage, offering a dietary approach to complement immunosuppressive therapies. Omega-3 polyunsaturated fatty acids (EPA, DHA) incorporated into enteral formulas reduce TLR4-mediated inflammatory responses to LPS released from pathogenic biofilms (93). Curcumin and resveratrol inhibit NF-κB activation, dampening cytokine storms (94, 95). Vitamin D3 enhances antimicrobial peptide production and supports regulatory T cell function, counteracting biofilm-induced inflammation (96, 97). Dietary fiber-derived butyrate promotes immune tolerance via GPR109A signaling (75).

**Figure 2:**
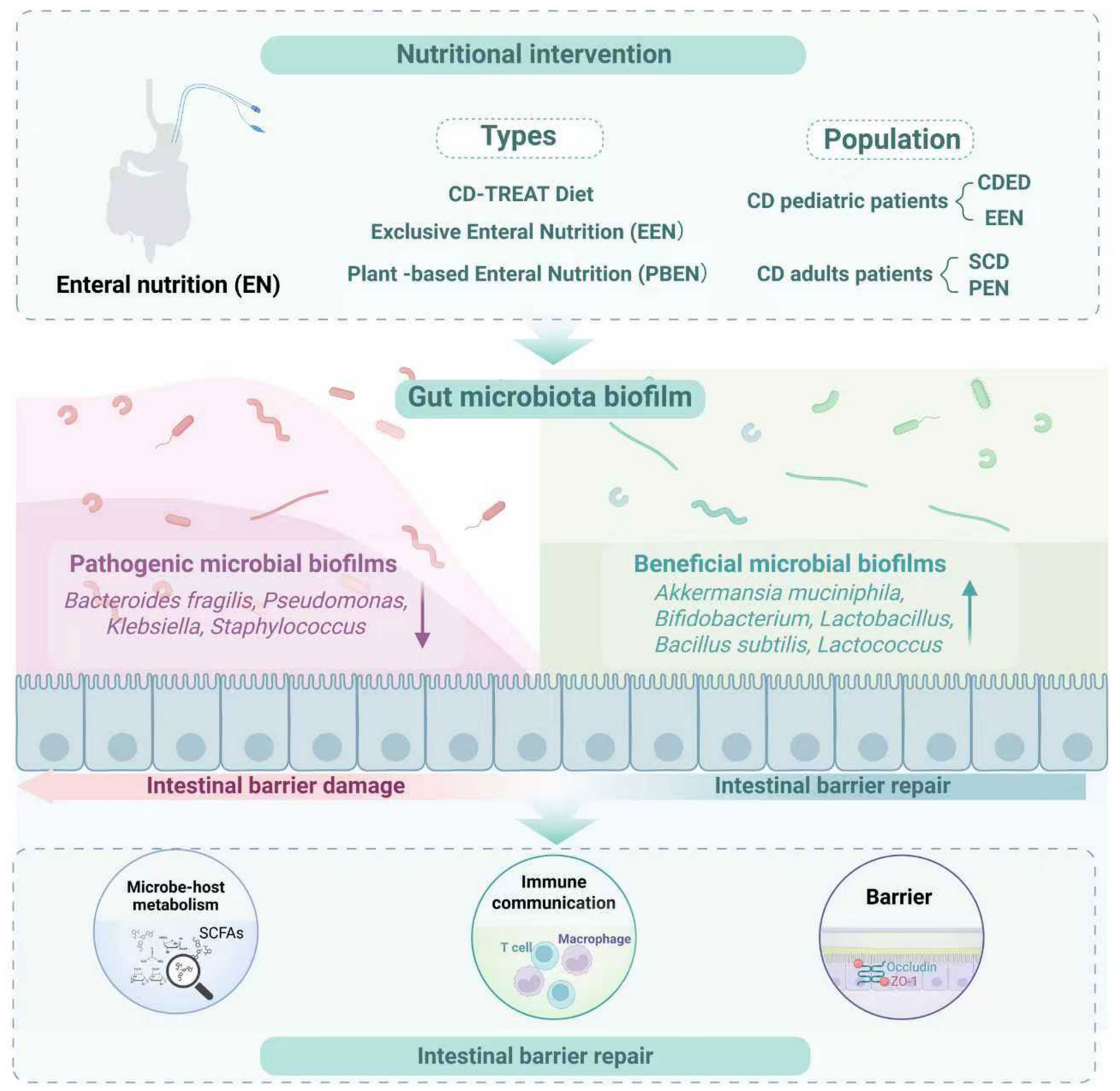
Dietary interventions modulate gut microbial biofilms and restore intestinal barrier function. Enteral nutrition remodels gut microbial biofilms by suppressing pathogenic bacteria and enriching beneficial microbes. The reshaped microbiota promotes intestinal barrier restoration via regulating microbial metabolism, immune crosstalk and epithelial tight junction integrity. Created in https://BioRender.com.

As a key interface for the interaction between intestinal microecology and the host, the structure and function of bacterial biofilm directly affect the integrity of the intestinal barrier. Nutrition intervention modulates the biofilm microenvironment through multiple targets—including optimizing the colonization of beneficial bacteria, inhibiting the formation of biofilms by pathogenic bacteria, and regulating the synthesis and distribution of metabolites (such as SCFAs and antimicrobial peptides), thereby reshaping the intestinal barrier function. This mechanism not only provides new ideas for nutritional treatment of diseases such as leaky gut syndrome and IBD but also lays a theoretical basis for ICU enteral nutritional support and application of probiotics/prebiotics (Figure 2).

## 3 Enteral nutrition targets biofilm via nutrient formulation

Nutritional interventions are increasingly recognized for their potential to modulate the gut microbiota, including the stability and composition of biofilms, as exemplified by strategies. Enteral nutrition (EN) directly nourishes intestinal flora by providing substrates such as dietary fiber and protein, promotes the formation of beneficial bacterial biofilms, and enhances intestinal barrier function. EN uses the function of the patient’s own digestive system to absorb nutrients in situations such as unable to take in adequate nutrients by mouth (e.g., short bowel syndrome, esophageal tumors) (98, 99), and diseases with basically normal gastrointestinal function (such as IBD) (100). EN modulates gut microbial biofilms can be categorized into three distinct but complementary strategies: (1) providing prebiotic substrates to promote beneficial biofilm formation; (2) restricting specific nutrients to suppress pathogenic biofilms; and (3) supplying specific amino acids or bioactive compounds that directly interfere with quorum sensing and virulence factor expression.

As shown in Table 3, three intestinal targeted intervention strategies are introduced: Crohn’s disease (CD)-TREAT diet, exclusive enteral nutrition (EEN) and plant-based enteral nutrition (PBEN). Specifically, the CD-TREAT diet operates primarily through nutrient restriction. By eliminating specific dietary components (such as emulsifiers and certain complex carbohydrates) that serve as substrates for pathogenic biofilm formation, CD-TREAT selectively suppresses the growth and biofilm integrity of detrimental bacteria like adherent-invasive *E. coli* (AIEC), while having minimal impact on beneficial commensals. This “subtraction strategy” has been shown to significantly reduce the concentration of pro-inflammatory markers and alter the metabolic landscape of the gut (101, 102). EEN represents an addition strategy that combines nutrient provision with direct barrier support. Beyond providing caloric needs, EEN contains specific components such as glutamine that directly enhance epithelial barrier function. Glutamine serves as a primary fuel source for enterocytes and promotes the expression of tight junction proteins (103). EEN increases the abundance of anaerobic SCFAs producers (e.g., Ruminococcaceae, Lachnospiraceae), which form porous, metabolically active biofilms that locally enrich butyrate and other barrier-protective metabolites. This dual action—direct epithelial support and indirect biofilm remodeling—underlies EEN’s therapeutic efficacy in Crohn’s disease (103). PBEN exemplifies a conversion strategy, wherein plant-derived fibers are fermented by specific bacterial groups to generate metabolites that reshape the biofilm environment. The predominance of Bacteroides species under PBEN is significant, as these bacteria possess extensive polysaccharide utilization loci that enable efficient fiber breakdown (104). The resulting SCFAs production, particularly butyrate, not only serves as an energy source for colonocytes but also creates a localized microenvironment that favors the maintenance of beneficial biofilm structures while suppressing pathogenic Enterobacteriaceae through pH reduction and competitive exclusion (104). These research results provide in-depth insights into the regulation of intestinal microecology in different groups: CD-TREAT and EEN show anti-inflammatory and metabolic regulatory value in the management of inflammatory bowel disease; symbiotic are suitable for improving intestinal dysfunction in elderly and long-term bedridden patients; PBEN provides a feasible strategy for the healthy reconstruction of intestinal flora and intervention of metabolic diseases in children. For long-term bedridden patients, nutritional intervention methods such as specific prebiotics and dietary fiber, combined with peristalsis promoters, can be used to target the structure and function of the intestinal flora biofilm to restore ecological balance. For long-term bedridden patients, regulating biofilms is a promising idea from “treating the symptoms” (changing the species) to “treating the root cause” (restoring ecosystem health). These three interventions illustrate distinct mechanistic approaches to biofilm modulation—subtraction (removing pro-pathogenic substrates), addition (providing direct barrier support and prebiotic substrates), and conversion (transforming dietary components into biofilm-modifying metabolites). The choice of strategy should be guided by the patient’s baseline biofilm profile and clinical presentation. Detailed information is shown in Table 3.

**Table 3:** Nutritional interventions targeting gut biofilms: mechanisms and clinical outcomes.

Intervention	Strategy type	Mechanism of biofilm modulation	Effects on microbiota	Biofilm-related metabolites	Clinical outcomes	Key Nutritional Components Related to Enteral Nutrition	References
CD-TREAT Diet	Nutrient restriction (subtraction)	↓Emulsifiers/complex carbohydrates (e.g., AIEC)	Not reported (shifts in pathogenic vs. beneficial balance inferred)	↓ Acetate, propionate, butyrate (reflecting reduced overall fermentation)	↓ Fecal calprotectin; ↓intestinal inflammation		(99, 100)
Exclusive enteral nutrition (EEN)	Nutrient addition + direct barrier support	↑ Glutamine; SCFAs	↑Ruminococcaceae, Lachnospiraceae	↑SCFAs (butyrate)	↓ Intestinal permeability; ↑ GLP-1/GLP-2; ↑glucose metabolism and insulin sensitivity		(103)
Plant-based enteral nutrition (PBEN)	Metabolic conversion	↑ Fermentable fibers; SCFAs	↑*Bacteroides*	↑ Fiber fermentation products, ↑ butyrate; ↓ pro-inflammatory bile acids	↑Energy, lipid, and glucose homeostasis; ↓pathogen colonization		(104)

These three interventions illustrate distinct mechanistic approaches to biofilm modulation—subtraction (removing pro-pathogenic substrates), addition (providing direct barrier support and prebiotic substrates), and conversion (transforming dietary components into biofilm-modifying metabolites). The choice of strategy should be guided by the patient’s baseline biofilm profile and clinical presentation.

The use of parenteral nutrition (PN) is essential to ensure appropriate nutritional intake and prevent malnutrition, which may lead to worsened outcomes. However, preclinical studies have shown that PN can cause atrophic damage to the intestinal mucosa and lead to considerable changes in intestinal microbial populations (105). The detrimental effects of PN on gut biofilms can be attributed to SCFA-producing bacteria (e.g., Faecalibacterium, Lachnospiraceae) reduction, mucosal atrophy, and immune dysregulation. This “hybrid nutrition” approach—combining PN with minimal enteral feeding—has shown promise in maintaining microbial diversity and SCFA production. Even small amounts of enteral substrate (as little as 10–20% of total caloric intake) may provide sufficient fermentable fiber to sustain baseline biofilm structure and function, preventing the complete ecological collapse associated with exclusive PN.

## 4 Nutritional intervention applications in IBD targeting bacterial biofilms

Given the availability of multiple nutritional interventions for IBD, clinicians face the challenge of selecting the most appropriate strategy for individual patients. We propose a biofilm-based clinical decision framework to guide this selection: (1) For patients with evidence of pathogenic biofilm overgrowth (e.g., elevated AIEC or *F. nucleatum* detected by stool or biopsy qPCR), interventions based on substrate deprivation (e.g., CDED) should be prioritized to remove substrates that support detrimental biofilm formation. (2) For patients with marked intestinal permeability (e.g., elevated lactulose/mannitol ratio), interventions combining direct barrier support with biofilm modulation (e.g., EEN) may offer promising therapeutic benefits through dual mechanisms. (3) For patients requiring long-term maintenance therapy, interventions with broad applicability and sustainability (e.g., SCD) may be most appropriate. (4) For patients unable to tolerate exclusive EN, partial enteral nutrition (PEN) provides a flexible alternative that can be combined with other dietary approaches. Building upon the biofilm-based clinical decision framework, Table 4 describes the mechanisms and clinical applications of nutritional intervention strategies targeting intestinal barrier, with a specific focus on bacterial biofilm modulation. These examples illustrate the translation of biofilm-targeted concepts into practical formulations: for pediatric CD patients with AIEC-predominant biofilms, CDED can be supplemented with mannose-rich oligosaccharides to compete with FimH-mediated adhesion and curcumin to disrupt AIEC biofilm matrix (106–110); for remission induction, EEN provides glutamine for epithelial repair and butyrate to locally enrich biofilm SCFAs (74, 111); for long-term maintenance, PEN incorporates FOS to stimulate beneficial biofilm-forming bacteria and lactoferrin to inhibit pathogen biofilm formation (66, 112); for broad-spectrum ecological modification, SCD can be enhanced with quercetin to inhibit Fusobacterium adhesion and cranberry proanthocyanidins to block pathogen biofilm integrity (113–115). These tailored formulations demonstrate how the choice of nutritional components can be guided by the predominant biofilm profile, moving beyond empirical supplementation toward biofilm-targeted nutrition. The choice among them should be guided by the patient’s individual characteristics: age (pediatric or adult), disease stage (active or remission), nutritional status, treatment history, and ideally, baseline biofilm profile (e.g., predominance of AIEC vs. *F. nucleatum* vs. protective species). These dietary strategies provide diverse, individualized, and mechanistically based nutritional solutions for the adjuvant treatment of CD, moving the field closer to the goal of individualized nutrition in IBD management.

**Table 4:** Nutritional interventions for IBD: mechanisms targeting bacterial biofilms.

Clinical States	Strategy type	Nutritional interventions	Nutritional components	Biofilm components	Microbiota and Metabolites	Biofilm-related outcomes	References
Pathogenic Biofilm Formation	Substrate deprivation (subtraction)	Crohn’s Disease Exclusion Diet (CDED)	Mannose-rich oligosaccharides; Curcumin	↓ Emulsifiers, ↓ complex carbohydrates (substrate for biofilm)	↑ *Lactobacillus*, ↑ *F. prausnitzii;* ↑ Lactic acid, ↑ butyric acid	↓Pathogenic biofilm density; ↑mucosal healing; ↑barrier integrity; ↓ NF-κB signaling	(83, 103, 105, 106)
Barrier dysfunction	Nutrient addition + direct barrier support	Exclusive Enteral Nutrition (EEN)	Glutamine; Butyrate	↓ Pro-inflammatory bacterial components (e.g., LPS)	↑ *Lactobacillus*, ↑ *Lachnospiraceae*; ↓ Enterobacteriaceae; ↑ SCFAs	↓ Intestinal permeability; ↓TNF-*α*-mediated barrier damage; ↑tight junction assembly	(107, 108)
Long-term Intervention	Maintenance modulation (hybrid)	Partial Enteral Nutrition (PEN)	FOS; Lactoferrin	↓ Glycolytic substrates for biofilm; ↑ AhR ligands	↑ *Lactobacillus*, ↑ *Bifidobacterium*, ↑ *Faecalibacterium; ↑ SCFAs, ↑ tryptophan metabolites*	↓ HDAC activity; ↓glycolysis and inflammation; ↑ M2 macrophage polarization	(46, 74, 109–111)
Critical care	Broad-spectrum ecological modification	The Specific Carbohydrate Diet (SCD)	Quercetin; Cranberry proanthocyanidins	↑ Complex carbohydrates (prebiotic), ↑ mucus production	↑ *F. prausnitzii*, ↑ *Lactobacillus*, ↑ *Bifidobacterium*, ↑ *A. muciniphila;* ↑ SCFAs, ↑ tryptophan metabolites	↑ Treg differentiation and expansion; ↑ Claudin and ZO-1 expression; ↑epithelial barrier integrity	(66, 112, 115)

These four interventions represent distinct mechanistic strategies for biofilm modulation in IBD. CDED operates through substrate deprivation (“subtraction”), EEN combines nutrient addition with direct barrier support (“addition + protection”), PEN provides maintenance modulation (“sustained support”), and SCD offers broad-spectrum ecological modification (“general remodeling”). Clinical selection should consider the patient’s age, disease stage, nutritional status, and underlying biofilm profile.

## 5 Future directions and recommendations

The essence of initial nutritional intervention, such as supplementing dietary fiber, prebiotics or probiotics, is the “macro-regulation” of the flora. “Regulating the number of bacteria” through nutritional means can indeed improve host physiological functions. A common confusion in clinical practice is the huge individual differences: the same probiotics or dietary regimen have significant effects on some patients, but have little effect on others, which reveals the necessity of going beyond the level of “bacterial species abundance” and delving into the interaction between bacterial community functions and ecology.

Based on these insights, we propose a Biofilm-Targeted Nutrition Framework that categorizes interventions into three functional classes: (1) Biofilm disruptors: Nutrients or compounds that specifically degrade pathogenic biofilm matrices (e.g., certain plant-derived enzymes, lactoferrin, specific polysaccharides that compete for adhesion sites). These are indicated when pathogenic biofilms predominate. (2) Biofilm enhancers: Substrates that promote EPS production and stabilize beneficial biofilms (e.g., specific prebiotic fibers that stimulate EPS synthesis, micronutrients that serve as cofactors for biofilm matrix production). These are indicated when beneficial biofilms are deficient. (3) Biofilm modulators: Nutrients that influence quorum sensing signals, shifting the competitive balance between beneficial and pathogenic communities (e.g., plant polyphenols that interfere with QS signaling, specific amino acids that modulate autoinducer production). These are indicated for long-term ecological maintenance. This framework provides a structured approach to designing nutritional interventions based on the patient’s biofilm profile, moving beyond the current practice of empirically selecting probiotics or prebiotics.

### 5.1 Addressing individual heterogeneity

Differences in individual flora are a key factor leading to variable responses to intervention. Individuals with poor baseline flora structure may be unresponsive and even suffer from gastrointestinal discomfort due to dysbiosis. This requires that future nutritional intervention must be personalized. It may be necessary to conduct bacterial genetic testing first, non-responders to fiber supplementation often lack specific fiber-degrading bacteria (e.g., *Ruminococcus bromii*, *B.thetaiotaomicron*), suggesting that these individuals may benefit from a sequential approach: first supplementing the missing degraders as probiotics, then providing the fiber substrate once the degraders are established. This “probiotic-prebiotic sequential therapy” represents a logical extension of the biofilm-centric view, recognizing that stable biofilms require both the appropriate microbial players and the nutritional substrates to sustain them.

### 5.2 Technological advances

To overcome the limitations of traditional metagenomics in analyzing the functional heterogeneity and spatial structure of bacterial biofilm, a series of cutting-edge technologies are being applied to nutrition research. By applying bacterial single-cell RNA sequencing, researchers found that after taking probiotics, only some bacteria showed high metabolic activity, while most were in a dormant or low-activity state, which might explain why the same probiotic has different effects in different individuals and whether biofilms formed or not (116). To solve the “spatial black box” problem of biofilm, high-resolution spatial transcriptomics technology emerged as the times require. For example, a study using high-throughput technologies (HTTs) on intestinal mucosal biopsies from patients with inflammatory bowel disease was the first to simultaneously analyze gene expression in the biofilm formation area and adjacent host epithelial cells *in situ*. It was found that host cells beneath the pathogenic biofilm highly expressed genes related to inflammation and apoptosis, while areas colonized by beneficial bacteria were enriched in barrier protection genes (117). This technology makes it possible to accurately correlate the “spatial positioning of bacterial colonies-host functional response.” These methods jointly promote nutrition intervention research from correlation analysis to causal bacterial biofilm analysis, providing a methodological basis for individualized nutrition in the future.

### 5.3 Biofilm-targeted clinical nutrition products

Potential products include biofilm-enhancing enteral formulas enriched with specific prebiotic fibers (e.g., FOS, GOS, HMOs) and micronutrients to promote beneficial biofilms like *Faecalibacterium* and *Akkermansia*; biofilm-disrupting functional foods incorporating lactoferrin, plant-derived enzymes, or polyphenol-rich extracts to target pathogenic biofilms; and personalized nutritional powders customized based on individual biofilm profiles. Food engineering technologies, such as microencapsulation to protect bioactive components during gastric transit, spray drying for powder stability, and nanoemulsion for enhanced bioavailability are essential to ensure targeted delivery to the intestinal mucosa. These product innovations, developed through close collaboration between microbiologists, clinical nutritionists, and food scientists, represent a new frontier in functional food development, offering concrete solutions for managing intestinal barrier dysfunction in conditions such as IBD and leaky gut syndrome.

## References

[1] Sanz Y Cryan JF Deschasaux-Tanguy M Elinav E Lambrecht R Veiga P. The gut microbiome connects nutrition and human health. Nat Rev Gastroenterol Hepatol. (2025) 22:534–55. doi: 10.1038/s41575-025-01077-5, 40468006

[2] Ray S Shankaran P. Nutrition and the gut microbiome: a symbiotic dialogue influencing health and disease. Front Nutr. (2026) 13:1761992. doi: 10.3389/fnut.2026.1761992, 41769655 PMC12935949

[3] Di Vincenzo F Del Gaudio A Petito V Lopetuso LR Scaldaferri F. Gut microbiota, intestinal permeability, and systemic inflammation: a narrative review. Intern Emerg Med. (2023) 19:275–93. doi: 10.1007/s11739-023-03374-w, 37505311 PMC10954893

[4] Jandl B Dighe S Baumgartner M Makristathis A Gasche C Muttenthaler M. Gastrointestinal biofilms: endoscopic detection, disease relevance, and therapeutic strategies. Gastroenterol. (2024) 167:1098–1112.e5. doi: 10.1053/j.gastro.2024.04.032, 38876174

[5] Wu Y Liu P Su Y Li H Geng Y Ren Y . In vitro evaluation of the intestinal probiotic effect of *Akkermansia muciniphila* ATCC BAA-835. Food Ferment Ind. (2022) 48:156–62. doi: 10.13995/j.cnki.11-1802/ts.027445

[6] Motta J-P Wallace JL Buret AG Deraison C Vergnolle N. Gastrointestinal biofilms in health and disease. Nat Rev Gastroenterol Hepatol. (2021) 18:314–34. doi: 10.1038/s41575-020-00397-y, 33510461

[7] Saharan BS Beniwal N Duhan JS. From formulation to function: a detailed review of microbial biofilms and their polymer-based extracellular substances. Microbe. (2024) 5:100194. doi: 10.1016/j.microb.2024.100194

[8] Ghosh SS Wang J Yannie PJ Ghosh S. Intestinal barrier function and metabolic/liver diseases. Liver Res. (2020) 4:81–7. doi: 10.1016/j.livres.2020.03.002

[9] Gong F Xin S Liu X He C Yu X Pan L . Multiple biological characteristics and functions of intestinal biofilm extracellular polymers: friend or foe? Front Microbiol. (2024) 15:1445630. doi: 10.3389/fmicb.2024.1445630, 39224216 PMC11367570

[10] Salehibarmi M Siroosi M. Effect of supplementation on biofilms and antibiotic efficacy against *Pseudomonas aeruginosa*. Iran J Microbiol. (2025) 17:861–74. doi: 10.18502/ijm.v17i6.20353, 41510040 PMC12777386

[11] Gao W Jing H Qiu B Zhang S Zhang J Xu L . Effects of biofilm formation on gastrointestinal tolerance, mucoadhesion and transcriptomic responses of probiotics. Food Sci Nutr. (2025) 13:e70206. doi: 10.1002/fsn3.70206, 40370418 PMC12076125

[12] Pérez-González A El Hajra I. Exclusive enteral nutrition as an effective treatment in inflammatory bowel disease. Recent Prog Nutr. (2025) 5:1–13. doi: 10.21926/rpn.2502010

[13] Fitzpatrick JA Melton SL Yao CK Gibson PR Halmos EP. Dietary management of adults with IBD — the emerging role of dietary therapy. Nat Rev Gastroenterol Hepatol. (2022) 19:652–69. doi: 10.1038/s41575-022-00619-5, 35577903

[14] Xu T Xiao Y Wang H Zhu J Lee Y Zhao J . Characterization of mixed-species biofilms formed by four gut microbiota. Microorganisms. (2022) 10:2332. doi: 10.3390/microorganisms10122332, 36557585 PMC9781930

[15] Li Y Rui W Sheng X Deng X Li X Meng L . *Bifidobacterium breve* synergizes with *Akkermansia muciniphila* and *Bacteroides ovatus* to antagonize *Clostridioides difficile*. ISME J. (2025) 19:wraf086. doi: 10.1093/ismejo/wraf086, 40302032 PMC12089032

[16] Béchon N Ghigo J-M. Gut biofilms: *Bacteroides* as model symbionts to study biofilm formation by intestinal anaerobes. FEMS Microbiol Rev. (2021) 46:fuab054. doi: 10.1093/femsre/fuab054, 34849798

[17] Jones SE Versalovic J. Probiotic *Lactobacillus reuteri* biofilms produce antimicrobial and anti-inflammatory factors. BMC Microbiol. (2009) 9:26. doi: 10.1186/1471-2180-9-26, 19210794 PMC2653509

[18] Yu J An N Cui Y Zhang L. Structural characterization and immunomodulatory effects of *Bifidobacterium bifidum* biofilms enriched with extracellular polysaccharides and surface proteins. Int J Biol Macromol. (2025) 328:147604. doi: 10.1016/j.ijbiomac.2025.147604, 40939802

[19] Duanis-Assaf D Steinberg D Chai Y Shemesh M. The LuxS based quorum sensing governs lactose induced biofilm formation by *Bacillus subtilis*. Front Microbiol. (2016) 6:1517. doi: 10.3389/fmicb.2015.01517, 26779171 PMC4705240

[20] Başaran SN. Postbiotic activity of *Akkermansia muciniphila* supernatant against antibiotic-resistant *Enterococcus faecalis*. Int Microbiol. (2025) 28:2821–8. doi: 10.1007/s10123-025-00733-9, 41068499

[21] Cui Y Wang D Zhang L Qu X. Research progress on the regulatory mechanism of biofilm formation in probiotic lactic acid bacteria. Crit Rev Food Sci Nutr. (2024) 65:4869–83. doi: 10.1080/10408398.2024.2400593, 39244761

[22] Pickard JM Zeng MY Caruso R Núñez G. Gut microbiota: role in pathogen colonization, immune responses and inflammatory disease. Immunol Rev. (2017) 279:70–89. doi: 10.1111/imr.12567, 28856738 PMC5657496

[23] Blanchett S Tsai CJ Sandford S Loh JM Huang L Kirman JR . Intranasal immunization with Ag85B peptide 25 displayed on *Lactococcus lactis* using the PilVax platform induces antigen-specific B- and T-cell responses. Immunol Cell Biol. (2021) 99:767–81. doi: 10.1111/imcb.12462, 33866609

[24] Vogt CM Hilbe M Ackermann M Aguilar C Eichwald C. Mouse intestinal microbiota reduction favors local intestinal immunity triggered by antigens displayed in *Bacillus subtilis* biofilm. Microb Cell Factories. (2018) 17:187. doi: 10.1186/s12934-018-1030-8, 30477481 PMC6258259

[25] Deryabin D Galadzhieva A Kosyan D Duskaev G. Plant-derived inhibitors of AHL-mediated quorum sensing in bacteria: modes of action. Int J Mol Sci. (2019) 20:5588. doi: 10.3390/ijms20225588, 31717364 PMC6888686

[26] Dong WW Zhu J Guo X Kong DL Zhang Q Zhou YQ . Characterization of AiiK, an AHL lactonase, from Kurthia huakui LAM0618T and its application in quorum quenching on *Pseudomonas aeruginosa* PAO1. Sci Rep. (2018) 8:6013. doi: 10.1038/s41598-018-24507-8, 29662232 PMC5902575

[27] Thananimit S Pahumunto N Teanpaisan R. Characterization of short chain fatty acids produced by selected potential probiotic *Lactobacillus* strains. Biomolecules. (2022) 12:1829. doi: 10.3390/biom12121829, 36551257 PMC9775007

[28] Chang KC Nagarajan N Gan YH. Short-chain fatty acids of various lengths differentially inhibit *Klebsiella pneumoniae* and *Enterobacteriaceae* species. mSphere. (2024) 9:e00781-23. doi: 10.1128/msphere.00781-23, 38305176 PMC10900885

[29] Lee YK Puong KY Ouwehand AC Salminen S. Displacement of bacterial pathogens from mucus and Caco-2 cell surface by lactobacilli. J Med Microbiol. (2003) 52:925–30. doi: 10.1099/jmm.0.05009-0, 12972590

[30] AlKhatib Z Lagedroste M Fey I Kleinschrodt D Abts A Smits SHJ. Lantibiotic immunity: inhibition of nisin mediated pore formation by NisI. PLoS One. (2014) 9:e102246. doi: 10.1371/journal.pone.0102246, 25014359 PMC4094520

[31] Scaffo J Durães Lima R Dobrotka C Ribeiro TAN Pereira RFA Sachs D . In vitro analysis of interactions between *Staphylococcus aureus* and *Pseudomonas aeruginosa* during biofilm formation. Antibiotics (Basel). (2025) 14:504. doi: 10.3390/antibiotics14050504, 40426570 PMC12108489

[32] Radlinski L Rowe SE Kartchner LB Maile R Cairns BA Vitko NP . *Pseudomonas aeruginosa* exoproducts determine antibiotic efficacy against *Staphylococcus aureus*. PLoS Biol. (2017) 15:e2003981. doi: 10.1371/journal.pbio.2003981, 29176757 PMC5720819

[33] Cangui Panchi SP Ñacato Toapanta AL Enríquez Martínez LJ Salinas Delgado GA Reyes J Garzon Chávez D . Battle royale: immune response on biofilms – host-pathogen interactions. Curr Res Immunol. (2023) 4:100057. doi: 10.1016/j.crimmu.2023.100057, 37025390 PMC10070391

[34] Qazi S Middleton B Muharram SH Cockayne A Hill P O'Shea P . N-Acylhomoserine lactones antagonize virulence gene expression and quorum sensing in *Staphylococcus aureus*. Infect Immun. (2006) 74:910–9. doi: 10.1128/IAI.74.2.910-919.2006, 16428734 PMC1360299

[35] Zhu H Liu HJ Ning SJ Gao YL. The response of type 2 quorum sensing in *Klebsiella pneumoniae* to a fluctuating culture environment. DNA Cell Biol. (2012) 31:455–9. doi: 10.1089/dna.2011.1375, 21877918

[36] Britigan BE Roeder TL Rasmussen GT Shasby DM McCormick ML Cox CD. Interaction of the *Pseudomonas aeruginosa* secretory products pyocyanin and pyochelin generates hydroxyl radical and causes synergistic damage to endothelial cells. J Clin Invest. (1992) 90:2187–96. doi: 10.1172/JCI116104, 1469082 PMC443369

[37] Pradhan D Tanwar A Wong J Parthasarathi S Frankel GM Singh V. Toroidal displacement of *Klebsiella pneumoniae* by *Pseudomonas aeruginosa* is a unique mechanism to avoid competition for iron. MBio. (2025) 16:e0114925. doi: 10.1128/MBIO.01149-25, 40497729 PMC12239573

[38] Zhou Y Chai Z Pandeya A Yang L Zhang Y Zhang G . Caspase-11 and NLRP3 exacerbate systemic Klebsiella infection through reducing mitochondrial ROS production. Front Immunol. (2025) 16:1516120. doi: 10.3389/fimmu.2025.1516120, 40034692 PMC11873083

[39] McGilligan VE Gregory-Ksander MS Li D Moore JE Hodges RR Gilmore MS . *Staphylococcus aureus* activates the NLRP3 inflammasome in human and rat conjunctival goblet cells. PLoS One. (2013) 8:e74010. doi: 10.1371/journal.pone.0074010, 24040145 PMC3769353

[40] Kaewarsar E Chaiyasut C Lailerd N Makhamrueang N Peerajan S Sirilun S. Optimization of mixed inulin, fructooligosaccharides, and galactooligosaccharides as prebiotics for stimulation of probiotics growth and function. Foods. (2023) 12:1591. doi: 10.3390/foods12081591, 37107386 PMC10137966

[41] Komatsu Y Furuichi M Kokubo T. Gastrointestinal journey of human milk oligosaccharides: from breastfeeding origins to functional roles in adults. Microorganisms. (2025) 14:29. doi: 10.3390/microorganisms14010029, 41597549 PMC12844028

[42] Yoshida K Kokubo E Morita S Sonoki H Miyaji K. Combination of inulin and resistant dextrin has superior prebiotic effects and reduces gas production during in vitro fermentation of fecal samples from older people. Nutrients. (2024) 16:4262. doi: 10.3390/nu16244262, 39770884 PMC11678394

[43] Lordan C Roche AK Delsing D Nauta A Groeneveld A MacSharry J . Linking human milk oligosaccharide metabolism and early life gut microbiota: bifidobacteria and beyond. Microbiol Mol Biol Rev. (2024) 88:e0009423. doi: 10.1128/mmbr.00094-23, 38206006 PMC10966949

[44] Ioannou P Baliou S. The molecular mechanisms and therapeutic potential of cranberry, D-mannose, and flavonoids against infectious diseases: the example of urinary tract infections. Antibiotics. (2024) 13:593. doi: 10.3390/antibiotics13070593, 39061275 PMC11273536

[45] Hao S Yang D Zhao L Shi F Ye G Fu H . EGCG-mediated potential inhibition of biofilm development and quorum sensing in *Pseudomonas aeruginosa*. Int J Mol Sci. (2021) 22:4946. doi: 10.3390/ijms22094946, 34066609 PMC8125375

[46] Tiwari R Tickell KD Yoshioka E Otieno J Shah A Richardson BA . Lactoferrin and lysozyme to promote nutritional, clinical and enteric recovery: a protocol for a factorial, blinded, placebo-controlled randomised trial among children with diarrhoea and malnutrition (the Boresha Afya trial). BMJ Open. (2024) 14:e079448. doi: 10.1136/bmjopen-2023-079448, 39122384 PMC11331881

[47] De Vos WM Nguyen Trung M Davids M Liu G Rios Morales M Jessen H . Phytate metabolism is mediated by microbial cross-feeding in the gut microbiota. Nat Microbiol. (2024) 9:1812–27. doi: 10.1038/s41564-024-01698-7, 38858593 PMC7618129

[48] Chen Y Gozzi K Yan F Chai YR. Acetic acid acts as a volatile signal to stimulate bacterial biofilm formation. mBio. (2015) 6:e00392. doi: 10.1128/mBio.00392-15, 26060272 PMC4462622

[49] Sakata T. Pitfalls in short-chain fatty acid research: a methodological review. Anim Sci J. (2019) 90:3–13. doi: 10.1111/asj.13118, 30370625 PMC6587520

[50] Gonçalves P Araújo JR Di Santo JP. A cross-talk between microbiota-derived short-chain fatty acids and the host mucosal immune system regulates intestinal homeostasis and inflammatory bowel disease. Inflamm Bowel Dis. (2018) 24:558–72. doi: 10.1093/ibd/izx029, 29462379

[51] Fernández-Pérez R Sáenz Y Rojo-Bezares B Zarazaga M Rodríguez JM Torres C . Production and antimicrobial activity of nisin under enological conditions. Front Microbiol. (2018) 9:1918. doi: 10.3389/fmicb.2018.01918, 30233504 PMC6134021

[52] Wang Y Li N Yang JJ Zhao DM Chen B Zhang GQ . Probiotics and fructo-oligosaccharide intervention modulate the microbiota-gut brain axis to improve autism spectrum reducing also the hyper-serotonergic state and the dopamine metabolism disorder. Pharmacol Res. (2020) 157:104784. doi: 10.1016/j.phrs.2020.104784, 32305492

[53] Jung D-H Park C-S. Resistant starch utilization by Bifidobacterium, the beneficial human gut bacteria. Food Sci Biotechnol. (2023) 32:441–52. Available at:. doi: 10.1007/s10068-023-01253-w, 36911330 PMC9992497

[54] Sasaki H Hayashi K Imamura M Hirota Y Hosoki H Nitta L . Combined resistant dextrin and low-dose Mg oxide administration increases short-chain fatty acid and lactic acid production by gut microbiota. J Nutr Biochem. (2023) 120:109420. doi: 10.1016/j.jnutbio.2023.109420, 37516314

[55] Liu N Chen B Zhao X Wen J Qi G. Cations and surfactin serving as signal molecules trigger quorum sensing in *Bacillus amyloliquefaciens*. J Basic Microbiol. (2022) 62:35–47. doi: 10.1002/jobm.202100315, 34825384

[56] Vastano V Pagano A Fusco A Merola G Sacco M Donnarumma G. The *Lactobacillus plantarum* Eno A1 enolase is involved in immunostimulation of Caco-2 cells and in biofilm development. Adv Exp Med Biol. (2016) 897:33–44. doi: 10.1007/5584_2015_5009, 26577529

[57] Man LL Xiang DJ. Effect of LuxS/AI-2-mediated quorum sensing system on bacteriocin production of *Lactobacillus plantarum* NMD-17. China Condiment. (2024) 68:855–66. doi: 10.1007/s12223-023-01060-0, 37156969

[58] Beisner J Filipe Rosa L Kaden-Volynets V Stolzer I Günther C Bischoff SC. Prebiotic inulin and sodium butyrate attenuate obesity-induced intestinal barrier dysfunction by induction of antimicrobial peptides. Front Immunol. (2021) 12:678360. doi: 10.3389/fimmu.2021.678360, 34177920 PMC8226265

[59] Joshi JR Khazanov N Charkowski A Faigenboim A Senderowitz H Yedidia I. Interkingdom signaling interference: the effect of plant-derived small molecules on quorum sensing in plant-pathogenic bacteria. Annu Rev Phytopathol. (2021) 59:153–90. doi: 10.1146/annurev-phyto-020620-095740, 33951403

[60] Yan S Chang J Hao X Liu J Tan X Geng Z . Berberine regulates short-chain fatty acid metabolism and alleviates the colitis-associated colorectal tumorigenesis through remodeling intestinal flora. Phytomedicine. (2022) 102:154217. doi: 10.1016/j.phymed.2022.154217, 35660350

[61] Munteanu C Onose G Rotariu M Poștaru M Turnea M Galaction AI. Role of microbiota-derived hydrogen sulfide (H2S) in modulating the gut–brain axis: implications for Alzheimer’s and Parkinson’s disease pathogenesis. Biomedicine. (2024) 12:2670. doi: 10.3390/biomedicines12122670, 39767577 PMC11727295

[62] Rhee SH. Lipopolysaccharide: basic biochemistry, intracellular signaling, and physiological impacts in the gut. Intest Res. (2014) 12:90–5. doi: 10.5217/ir.2014.12.2.90, 25349574 PMC4204704

[63] Szabo C. Hydrogen sulfide, an endogenous stimulator of mitochondrial function in cancer cells. Cells. (2021) 10:220. doi: 10.3390/cells10020220, 33499368 PMC7911547

[64] Upadhyay A Pal D Kumar A. Combinatorial enzyme therapy: a promising neoteric approach for bacterial biofilm disruption. Process Biochem. (2023) 129:56–66. doi: 10.1016/j.procbio.2023.02.022

[65] Hengge R. Targeting bacterial biofilms by the Green tea polyphenol EGCG. Molecules. (2019) 24:2403. doi: 10.3390/molecules24132403, 31261858 PMC6650844

[66] de Sá Almeida JS de Oliveira Marre AT Teixeira FL Boente RF Domingues RMCP de Paula GR . Lactoferrin and lactoferricin B reduce adhesion and biofilm formation in the intestinal symbionts Bacteroides fragilis and *Bacteroides thetaiotaomicron*. Anaerobe. (2020) 64:102232. doi: 10.1016/j.anaerobe.2020.102232, 32634470

[67] Prabu SM Shagirtha K Renugadevi J. Quercetin in combination with vitamins (C and E) improve oxidative stress and hepatic injury in cadmium intoxicated rats. Biomed Prev Nutr. (2011) 1:1–7. doi: 10.1016/j.bionut.2010.12.003

[68] Suzuki T. Regulation of the intestinal barrier by nutrients: the role of tight junctions. Anim Sci J. (2020) 91:e13357. doi: 10.1111/asj.13357, 32219956 PMC7187240

[69] Zhou X Qi W Hong T Xiong T Gong D Xie M . Exopolysaccharides from *Lactobacillus plantarum* NCU116 regulate intestinal barrier function via STAT3 signaling pathway. J Agric Food Chem. (2018) 66:9719–27. doi: 10.1021/acs.jafc.8b03340, 30134660

[70] Ishikawa H Kuno Y Yokoo T Nagashima R Takaki T Sasaki H . In vitro investigation of the effects of *Lactobacillus delbrueckii* ssp. bulgaricus OLL1073R-1 exopolysaccharides on tight junction damage caused by influenza virus infection. Lett Appl Microbiol. (2024) 77:ovae029. doi: 10.1093/lambio/ovae029, 38521981

[71] Wachi S Kanmani P Tomosada Y Kobayashi H Yuri T Egusa S . *Lactobacillus delbrueckii* TUA4408L and its extracellular polysaccharides attenuate enterotoxigenic *Escherichia coli*-induced inflammatory response in porcine intestinal epitheliocytes via toll-like receptor-2 and 4. Mol Nutr Food Res. (2014) 58:2080–93. doi: 10.1002/mnfr.201400218, 24995380

[72] Lin S Deng D Buskermolen JK Roffel S Janus MM Krom BP . Commensal and pathogenic biofilms alter toll-like receptor signaling in reconstructed human gingiva. Front Cell Infect Microbiol. (2019) 9:282. doi: 10.3389/fcimb.2019.00282, 31448244 PMC6692492

[73] Beutheu S Ghouzali I Galas L Déchelotte P Coëffier M. Glutamine and arginine improve permeability and tight junction protein expression in methotrexate-treated Caco-2 cells. Clin Nutr. (2013) 32:863–9. doi: 10.1016/j.clnu.2013.01.014, 23428392

[74] Kuo Y Lin CH Lin WS Pan MH. L-glutamine substantially improves 5-fluorouracil-induced intestinal mucositis by modulating gut microbiota and maintaining the integrity of the gut barrier in mice. Mol Nutr Food Res. (2024) 68:e2300704. doi: 10.1002/mnfr.202300704, 38656560

[75] Deng G Wen B Jia L Liu J Yan Q. *Clostridium butyricum* upregulates GPR109A/AMPK/PGC-1α and ameliorates acute pancreatitis-associated intestinal barrier injury in mice. Arch Microbiol. (2024) 206:04001–8. doi: 10.1007/s00203-024-04001-8, 38761195

[76] Harrer A Bücker R Boehm M Zarzecka U Tegtmeyer N Sticht H . *Campylobacter jejuni* enters gut epithelial cells and impairs intestinal barrier function through cleavage of occludin by serine protease HtrA. Gut Pathog. (2019) 11:4. doi: 10.1186/s13099-019-0283-z, 30805031 PMC6373145

[77] Butt S Saleh M Gagnon J. Impact of the *Escherichia coli* heat-stable enterotoxin b (STb) on gut health and function. Toxins. (2020) 12:760. doi: 10.3390/toxins12120760, 33276476 PMC7761119

[78] Tiwary BK Adhikari MD. Targeting quorum sensing as a next-generation approach to bacterial infections. Open Access J Microbiol Biotechnol. (2024) 9:1–6. doi: 10.23880/oajmb-16000307, 42269566

[79] Rodrigues DF Elimelech M. Role of type 1 fimbriae and mannose in the development of *Escherichia Coli* K12 biofilm: from initial cell adhesion to biofilm formation. Biofouling. (2009) 25:401–11. doi: 10.1080/08927010902833443, 19306144

[80] Yan QQ Jia L Wen B Wu Y Zeng Y Wang Q. *Clostridium Butyricum* protects against pancreatic and intestinal injury after severe acute pancreatitis via downregulation of MMP9. Front Pharmacol. (2022) 13:919010. doi: 10.3389/fphar.2022.919010, 35924043 PMC9342915

[81] Huang L Cao C Lin X Lu L Lin X Liu HC . Zinc alleviates thermal stress-induced damage to the integrity and barrier function of cultured chicken embryonic primary Jejunal epithelial cells via the MAPK and PI3K/AKT/mTOR signaling pathways. Poult Sci. (2024) 103:103696. doi: 10.1016/j.psj.2024.103696, 38593549 PMC11016803

[82] Gupta S Sarangi PP. Inflammation driven metabolic regulation and adaptation in macrophages. Clin Immunol. (2023) 246:109216. doi: 10.1016/j.clim.2022.109216, 36572212

[83] Wrzosek L Miquel S Noordine ML Bouet S Joncquel Chevalier-Curt M Robert V . *Bacteroides thetaiotaomicron* and *Faecalibacterium prausnitzii* influence the production of mucus glycans and the development of goblet cells in the colonic epithelium of a gnotobiotic model rodent. BMC Biol. (2013) 11:61. doi: 10.1186/1741-7007-11-61, 23692866 PMC3673873

[84] Yu J Wang S Yuan H Qiao T Bao M. Expression of Th17/Treg cells in peripheral blood and related cytokines of patients with ulcerative colitis of different syndrome types and correlation with the disease. Evid Based Complement Alternat Med. (2021) 2021:1–7. doi: 10.1155/2021/4600947, 34603468 PMC8486524

[85] Mohebali N Weigel M Hain T Sütel M Bull J Kreikemeyer B . *Faecalibacterium prausnitzii*, Bacteroides faecis and *Roseburia intestinalis* attenuate clinical symptoms of experimental colitis by regulating Treg/Th17 cell balance and intestinal barrier integrity. Biomed Pharmacother. (2023) 167:115568. doi: 10.1016/j.biopha.2023.115568, 37793274

[86] Wang G Fan Y Zhang G Cai S Ma Y Yang L . Microbiota-derived indoles alleviate intestinal inflammation and modulate microbiome by microbial cross-feeding. Microbiome. (2024) 12:59. doi: 10.1186/s40168-024-01750-y, 38504383 PMC10949743

[87] Wang M Guo J Hart AL Li JV. Indole-3-aldehyde reduces inflammatory responses and restores intestinal epithelial barrier function partially via aryl hydrocarbon receptor (AhR) in experimental colitis models. J Inflamm Res. (2023) 16:5845–64. doi: 10.2147/jir.s432747, 38084103 PMC10710743

[88] Sun D Zhou X Wu T Li Z Huang S Peng Z. Quercetin promotes the M1-to-M2 macrophage phenotypic switch during liver fibrosis treatment by modulating the JAK2/STAT3 signaling pathway. Recent Pat Anticancer Drug Discov. (2026) 21:31–46. doi: 10.3389/fphar.2024.1516609, 39360530

[89] Wang P Li Z Song Y Zhang B Fan C. Resveratrol-driven macrophage polarization: unveiling mechanisms and therapeutic potential. Front Pharmacol. (2025) 15:151660. doi: 10.3389/fphar.2024.151660 PMC11770351 39872049

[90] Chen B Guan L Wu C Gong Y Wu L Zhang M . Gut microbiota-butyrate-PPARγ Axis modulates adipose regulatory T cell population. Adv Sci. (2025) 12:20. doi: 10.1002/advs.202411086, 39998325 PMC12120792

[91] Planeta Kepp K. Bioinorganic chemistry of zinc in relation to the immune system. Chembiochem. (2022) 23:e202100554. doi: 10.1002/cbic.202100554, 34889510

[92] Nabhani ZA Dietrich G Hugot JP Barreau F. Nod2: the intestinal gate keeper. PLoS Pathog. (2017) 13:e1006177. doi: 10.1371/journal.ppat.1006177, 28253332 PMC5333895

[93] Singer P Bendavid I Mesilati-Stahy R Green P Rigler M Lev S . Enteral and supplemental parenteral nutrition enriched with omega-3 polyunsaturated fatty acids in intensive care patients – a randomized, controlled, double-blind clinical trial. Clin Nutr. (2021) 40:2544–54. doi: 10.1016/j.clnu.2021.03.034, 33932802

[94] Wang SL Li Y Wen Y Chen YF Na LX Li ST . Curcumin, a potential inhibitor of up-regulation of TNF-alpha and IL-6 induced by palmitate in 3T3-L1 adipocytes through NF-kappaB and JNK pathway. Biomed Environ Sci. (2009) 22:32–9. doi: 10.1016/s0895-3988(09)60019-2, 19462685

[95] Csaki C Mobasheri A Shakibaei M. Synergistic chondroprotective effects of curcumin and resveratrol in human articular chondrocytes: inhibition of IL-1β-induced NF-κB-mediated inflammation and apoptosis. Arthritis Res Ther. (2009) 11:R165. doi: 10.1186/ar2850, 19889203 PMC3003513

[96] Arliny Y Hasan M. Cathelicidin, vitamin D3 dan imunitas terhadap tuberkulosis. J Kedokt Syiah Kuala. (2020) 20:3. doi: 10.24815/jks.v20i3.18610

[97] Asgharp P. Eftekhari Z. Nadealian M. G. Borujeni G. N. Dezfouli M. R. M. Effect of Active vitamin D3 On the Expression of Antimicrobial Peptide Genes in Experimental Calf Pneumonia (2021). Urmia, Iran: Urmia University.

[98] Zeng H Zhao W Zhang X Wang X Luo P Li H . How enteral nutrition modes influence nasopharyngeal carcinoma survivors with late dysphagia: a randomized controlled study. Support Care Cancer. (2024) 32:702. doi: 10.1007/s00520-024-08912-6, 39367230

[99] Axelrod D Kazmerski K Iyer K. Pediatric enteral nutrition. JPEN J Parenter Enteral Nutr. (2006) 30:S21–6. doi: 10.1177/01486071060300S1S21, 16387906

[100] Triantafillidis JK Vagianos C Papalois AE. The role of enteral nutrition in patients with inflammatory bowel disease: current aspects. Biomed Res Int. (2015) 2015:197167. doi: 10.1155/2015/197167, 25793189 PMC4352452

[101] Levine A Wine E Assa A Boneh RS Shaoul R Kori M . Crohn’s disease exclusion diet plus partial enteral nutrition induces sustained remission in a randomized controlled trial. Gastroenterology. (2019) 157:440–450.e8. doi: 10.1053/j.gastro.2019.04.021, 31170412

[102] Svolos V Hansen R Nichols B Quince C Ijaz UZ Papadopoulou RT . Treatment of active Crohn’s disease with an ordinary food-based diet that replicates exclusive enteral nutrition. Gastroenterol. (2019) 156:1354–1367.e6. doi: 10.1053/j.gastro.2018.12.002, 30550821

[103] Li G Wu X Gao X Lin R Chen L Sun M . Long-term exclusive enteral nutrition remodels the gut microbiota and alleviates TNBS-induced colitis in mice. Food Funct. (2022) 13:1725–40. doi: 10.1039/D1FO03579G, 35085377

[104] Yeh A Conners EM Ramos-Jimenez RG Firek B Novak EA Rogers MB . Plant-based enteral nutrition modifies the gut microbiota and improves outcomes in murine models of colitis. Cell Mol Gastroenterol Hepatol. (2019) 7:872–874.e6. doi: 10.1016/j.jcmgh.2019.01.007, 30716421 PMC6522637

[105] Miyasaka EA Feng Y Poroyko V Falkowski NR Erb-Downward J Gillilland MG . Total parenteral nutrition–associated lamina propria inflammation in mice is mediated by a MyD88-dependent mechanism. J Immunol. (2013) 190:6607–15. doi: 10.4049/jimmunol.1201746, 23667106 PMC3679213

[106] Chevalier G Laveissière A Desachy G Barnich N Sivignon A Maresca M . Blockage of bacterial FimH prevents mucosal inflammation associated with Crohn’s disease. Microbiome. (2021) 9:1. doi: 10.1186/s40168-021-01135-5, 34425887 PMC8383459

[107] Mydock-McGrane LK Cusumano ZT Janetka JW. Mannose-derived FimH antagonists: a promising anti-virulence therapeutic strategy for urinary tract infections and Crohn’s disease. Expert Opin Ther Pat. (2016) 26:175–97. doi: 10.1517/13543776.2016.1131266, 26651364

[108] Packiavathy IASV Packiavathy IA Priya S Pandian SK Ravi AV. Inhibition of biofilm development of uropathogens by curcumin – an anti-quorum sensing agent from *Curcuma longa*. Food Chem. (2014) 148:453–60. doi: 10.1016/j.foodchem.2012.08.002, 24262582

[109] Verburgt CM Ghiboub M Benninga MA de Jonge WJ Van Limbergen JE. Nutritional therapy strategies in pediatric Crohn’s disease. Nutrients. (2021) 13:212. doi: 10.3390/nu13010212, 33450982 PMC7828385

[110] Palmela C Chevarin C Xu Z Torres J Sevrin G Hirten R . Adherent-invasive *Escherichia coli* in inflammatory bowel disease. Gut. (2018) 67:574–87. doi: 10.1136/gutjnl-2017-314903, 29141957

[111] Zhu M Lu EQ Yan L Liu G Huang K Xu E . Phospholipase D mediates glutamine-induced mTORC1 activation to promote porcine intestinal epithelial cell proliferation. J Nutr. (2024) 154:1119–29. doi: 10.1016/j.tjnut.2024.02.010, 38365119

[112] Yap BJM Yeo SK Ng WK Yong PVC Teo A Chua CLL. Fructooligosaccharides and immune health: immunomodulatory effects via gut microbiota and direct molecular mechanisms. Int J Food Sci Nutr. (2025) 76:675–88. doi: 10.1080/09637486.2025.256867, 41053937

[113] D’Arrigo M Muscarà C Molonia MS Cimino F Gervasi T. Synbiotic effect of quercetin and probiotic *Lactobacillus* SP. protects intestinal barrier from *E. coli*-induced challenge in Caco-2 cells. J Funct Foods. (2024) 114:106062. doi: 10.1016/j.jff.2024.106062, 38826717

[114] Alfaro-Viquez E Esquivel-Alvarado D Madrigal-Carballo S Krueger CG Reed JD. Proanthocyanidin-chitosan composite nanoparticles prevent bacterial invasion and colonization of gut epithelial cells by extra-intestinal pathogenic *Escherichia coli*. Int J Biol Macromol. (2019) 135:630–6. doi: 10.1016/j.ijbiomac.2019.04.170, 31128185

[115] Damas OM Garces L Abreu MT. Diet as adjunctive treatment for inflammatory bowel disease: review and update of the latest literature. Curr Treat Options Gastroenterol. (2019) 17:313–25. doi: 10.1007/s11938-019-00231-8, 30968340 PMC6857843

[116] Zmora N Zilberman-Schapira G Suez J Mor U Dori-Bachash M Bashiardes S . Personalized gut mucosal colonization resistance to empiric probiotics is associated with unique host and microbiome features. Cell. (2018) 174:1388–1405.e21. doi: 10.1016/j.cell.2018.08.041, 30193112

[117] Xu C Shao J. High-throughput omics technologies in inflammatory bowel disease. Clin Chim Acta. (2024) 555:117828. doi: 10.1016/j.cca.2024.117828, 38355001

[118] Shen Y Giardino Torchia ML Lawson GW Karp CL Ashwell JD Mazmanian SK. Outer membrane vesicles of a human commensal mediate immune regulation and disease protection. Cell Host Microbe. (2012) 12:509–20. doi: 10.1016/j.chom.2012.08.004, 22999859 PMC3895402

[119] Carrillo Terrazas M Oles R Hsu CY Loomis L Chu H. Strain-variation within *Bacteroides fragilis* species shapes immunomodulatory functions. J Immunol. (2024) 212:0708-7670. doi: 10.4049/jimmunol.212.1.Supplement.0708_7670

[120] Tebyanian H Bakhtiari A Karami A Kariminik A. Antimicrobial activity of some *Lactobacillus* species against intestinal pathogenic bacteria. Int Lett Nat Sci. (2017) 65:10–5. doi: 10.56431/p-c620g7, 42352281

[121] Tenea GN Garzón K Barrigas A Ortega C. Antimicrobial peptides of *Lactobacillus plantarum* UTNCys3.4 isolated from native fruits of Ecuadorian Amazonia inhibit the growth of foodborne pathogens. Mol2Net. (2017):3867. doi: 10.3390/mol2net-02-03867

[122] Ventolini G Mitchell E Salazar M. Biofilm formation by vaginal *Lactobacillus* in vivo. Med Hypotheses. (2015) 84:417–20. doi: 10.1016/j.mehy.2014.12.020, 25725906

[123] Qin D Ma Y Wang Y Hou X Yu L. Contribution of lactobacilli on intestinal mucosal barrier and diseases: perspectives and challenges of *Lactobacillus casei*. Life. (2022) 12:1910. doi: 10.3390/life12111910, 36431045 PMC9696601

[124] Cazorla SI Maldonado-Galdeano C Weill R De Paula J Perdigón GDV. Oral administration of probiotics increases Paneth cells and intestinal antimicrobial activity. Front Microbiol. (2018) 9:736. doi: 10.3389/fmicb.2018.00736, 29713315 PMC5911494

[125] Shen X Xie A Li Z Jiang C Wu J Li M . Research progress for probiotics regulating intestinal flora to improve functional dyspepsia: a review. Foods. (2024) 13:151. doi: 10.3390/foods13010151, 38201179 PMC10778471

[126] Arnaouteli S Bamford NC Stanley-Wall NR Kovács ÁT. *Bacillus subtilis* biofilm formation and social interactions. Nat Rev Microbiol. (2021) 19:600–14. doi: 10.1038/s41579-021-00540-9, 33824496

[127] Chai Y Chu F Kolter R Losick R. Bistability and biofilm formation in *Bacillus subtilis*. Mol Microbiol. (2008) 67:254–63. doi: 10.1111/j.1365-2958.2007.06040.x, 18047568 PMC2430929

[128] Luo H Wan K Wang HH. High-frequency conjugation system facilitates biofilm formation and pAMβ1 transmission by *Lactococcus lactis*. Appl Environ Microbiol. (2005) 71:2970–8. doi: 10.1128/AEM.71.6.2970-2978.2005, 15932992 PMC1151824

[129] Fanning S Hall LJ van Sinderen D. *Bifidobacterium breve* UCC2003 surface exopolysaccharide production is a beneficial trait mediating commensal-host interaction through immune modulation and pathogen protection. Gut Microbes. (2012) 3:420–5. doi: 10.4161/gmic.20630, 22713271

[130] Mandelbaum N Zhang L Carasso S Ziv T LifshizSimon S Davidovich I . Extracellular vesicles of the gram-positive gut symbiont *Bifidobacterium longum* induce immune-modulatory, anti-inflammatory effects. NPJ Biofilms Microbiomes. (2023) 9:30. doi: 10.1038/s41522-023-00400-9, 37270554 PMC10239484

[131] Hidalgo-Cantabrana C Sánchez B Milani C Ventura M Margolles A Ruas-Madiedo P. Genomic overview and biological functions of exopolysaccharide biosynthesis in Bifidobacterium spp. Appl Environ Microbiol. (2014) 80:9–18. doi: 10.1128/AEM.02977-13, 24123746 PMC3910989

[132] Laiño J Villena J Kanmani P Kitazawa H. Immunoregulatory effects triggered by lactic acid bacteria exopolysaccharides: new insights into molecular interactions with host cells. Microorganisms. (2016) 4:27. doi: 10.3390/microorganisms4030027, 27681921 PMC5039587

[133] Liu Z Li L Fang Z Lee Y Zhao J Zhang H . Integration of transcriptome and metabolome reveals the genes and metabolites involved in *Bifidobacterium bifidum* biofilm formation. Int J Mol Sci. (2021) 22:7596. doi: 10.3390/ijms22147596, 34299216 PMC8304991

[134] Zhang T Liu Z Wang H Zhang H Li H Lu W . Multi-omics analysis reveals genes and metabolites involved in *Bifidobacterium pseudocatenulatum* biofilm formation. Front Microbiol. (2023) 14:1287680. doi: 10.3389/fmicb.2023.1287680, 38029154 PMC10666050

[135] Li H He B Ma N Liu C Cai K Zhang X . Quorum sensing of Bifidobacteria: research and progress. Microbiol Res. (2025) 294:128102. doi: 10.1016/j.micres.2025.128102, 39965277

[136] Ding Y Hou Y Lao X. The role of *Akkermansia muciniphila* in disease regulation. Probiotics Antimicrob Proteins. (2025) 17:2027–38. doi: 10.1007/s12602-025-10642-y, 40632459

[137] Cani PD de Vos WM. Next-generation beneficial microbes: the case of *Akkermansia muciniphila*. Front Microbiol. (2017) 8:1765. doi: 10.3389/fmicb.2017.01765, 29018410 PMC5614963

[138] Solano C Echeverz M Lasa I. Biofilm dispersion and quorum sensing. Curr Opin Microbiol. (2014) 18:96–104. doi: 10.1016/j.mib.2014.02.008, 24657330

[139] Wang X Liu M Yu C Li J Zhou X. Biofilm formation: mechanistic insights and therapeutic targets. Mol Biomed. (2023) 4:49. doi: 10.1186/s43556-023-00164-w, 38097907 PMC10721784

[140] Bhattacharya S Mukherjee S Bandyopadhyay A. In vitro biofilm formation kinetics of *Pseudomonas aeruginosa* and *Escherichia coli* on medical-grade polyether ether ketone (PEEK) and polyamide 12 (PA12) polymers. Hygie. (2023) 5:32. doi: 10.3390/HYGIENE5030032, 30654563

[141] López CM García RT. "Bacterial biofilm formation and immune evasion mechanisms". In: Johnson MA Smith KL, editors. The human Microbiota and Chronic Disease. Lausanne: John Wiley & Sons, Inc (2022). p. 143–67.

[142] Liu C Sun D Zhu J Liu J Liu W. The regulation of bacterial biofilm formation by cAMP-CRP: a mini-review. Front Microbiol. (2020) 11:802. doi: 10.3389/fmicb.2020.00802, 32528421 PMC7247823

[143] Li X Li J Wu W Wu X Ren J. *Klebsiella pneumoniae* capsular polysaccharide: mechanism in regulation of synthesis, virulence, and pathogenicity. Virulence. (2024) 15:2439509. doi: 10.1080/21505594.2024.2439509, 39668724 PMC11649230

[144] Murphy CN Clegg S. Klebsiella pneumoniae and type 3 fimbriae: nosocomial infection, regulation and biofilm formation. Future Microbiol. (2012) 7:991–1002. doi: 10.2217/fmb.12.74, 22913357

[145] Liu JR Chen BX Jiang MT Cui TY Lv B Fu ZF . Polygonatum odoratum polysaccharide attenuates lipopolysaccharide-induced lung injury in mice by regulating gut microbiota. Food Sci Nutr. (2023) 11:6974–86. doi: 10.1002/fsn3.3622, 37970373 PMC10630852

[146] Li B Zhao Y Liu C Chen Z Zhou D. Molecular pathogenesis of *Klebsiella pneumoniae*. Future Microbiol. (2014) 9:1071–81. doi: 10.2217/fmb.14.48, 25340836

[147] Li Y Ni M. Regulation of biofilm formation in *Klebsiella pneumoniae*. Front Microbiol. (2023) 14:1238482. doi: 10.3389/fmicb.2023.1238482, 37744914 PMC10513181

[148] Chen L Wilksch JJ Liu H Zhang X Torres VVL Bi W . Investigation of LuxS-mediated quorum sensing in *Klebsiella pneumoniae*. J Med Microbiol. (2020) 69:402–13. doi: 10.1099/jmm.0.001148, 32223838 PMC7377169

[149] Peng Q Tang X Dong W Sun N Yuan W. A review of biofilm formation of *Staphylococcus aureus* and its regulation mechanism. Antibiotics (Basel). (2022) 12:12. doi: 10.3390/antibiotics12010012, 36671212 PMC9854888

[150] Wu X Wang H Xiong J Yang GX Hu JF Zhu Q . *Staphylococcus aureus* biofilm: formulation, regulatory, and emerging natural products-derived therapeutics. Biofilms. (2024) 7:100175. doi: 10.1016/j.bioflm.2023.100175, 38298832 PMC10827693

[151] Pumbwe L Skilbeck CA Wexler HM. Presence of quorum-sensing systems associated with multidrug resistance and biofilm formation in *Bacteroides fragilis*. Microb Ecol. (2008) 56:412–9. doi: 10.1007/s00248-007-9358-3, 18188535

[152] Yekani M Baghi HB Vahed SZ Ghanbari H Hosseinpur R Azargun R . Tightly controlled response to oxidative stress; an important factor in the tolerance of *Bacteroides fragilis*. Res Microbiol. (2021) 172:103798. doi: 10.1016/j.resmic.2021.103798, 33485914

[153] Delcaru C Alexandru I Podgoreanu P Grosu M Stavropoulos E Chifiriuc MC . Microbial biofilms in urinary tract infections and prostatitis: etiology, pathogenicity, and combating strategies. Pathogens. (2016) 5:65. doi: 10.3390/pathogens5040065, 27916925 PMC5198165

